# Editorial: Neural Prostheses for Locomotion

**DOI:** 10.3389/fnins.2021.788021

**Published:** 2021-11-04

**Authors:** Yury Ivanenko, Daniel P. Ferris, Kyuhwa Lee, Yoshio Sakurai, Irina N. Beloozerova, Mikhail Lebedev

**Affiliations:** ^1^Laboratory of Neuromotor Physiology, IRCCS Fondazione Santa Lucia, Rome, Italy; ^2^V. Zelman Center for Neurobiology and Brain Restoration, Skolkovo Institute of Science and Technology, Moscow, Russia; ^3^J. Crayton Pruitt Family Department of Biomedical Engineering, University of Florida, Gainesville, FL, United States; ^4^Wyss Center for Bio and Neuroengineering, Genève, Switzerland; ^5^Graduate School of Brain Science, Doshisha University, Kyoto, Japan; ^6^School of Biological Sciences, Georgia Institute of Technology, Atlanta, GA, United States; ^7^Center for Bioelectric Interfaces, National Research University Higher School of Economics, Moscow, Russia

**Keywords:** locomotion, exoskeletons, neuromodulation, brain computer interface, EEG, EMG, locomotor-related disorders, neurorehabilitation

During the last decade, we have seen a rapid development of neural prostheses, the systems that connect the brain to external assistive and rehabilitation devices. While this work was dominated by the research on neural prostheses that enable sensorimotor functions of the arm and hand, there has also been a growing interest in neural prostheses that restore locomotion, the ability to move in space. Brain-controlled wheelchairs and exoskeletons represent examples of such neural prostheses.

The collection of articles in this Research Topic present and discuss available evidence, conceptual frameworks, neuroprosthetic designs and practical questions concerning application of neural prostheses to gait assistance and rehabilitation. The Research Topic covers a range of issues such as control schemes, robotic aspects, effectiveness, characteristics of motor performance, neural underpinnings, ethics, significance for neurological disorders, motor learning, and recovery of locomotor function. The contributions are summarized below in 7 thematic categories: (i) reviews and perspectives, (ii) animal studies, (iii) balance control, (iv) prostheses for locomotion, (v) myoelectric control, (vi) electroencephalography (EEG)-based systems for lower limb prosthesis control, and (vii) combined effects of spinal cord neuromodulation and exoskeleton gait training in paralyzed individuals.

## Reviews and Perspectives

Two review articles included in the Research Topic assess the current state of assistive devices for locomotion and their perspectives for development. The review by Tariq et al. discusses a number of key features of the EEG-based activity mode recognition and the potentials of EEG-based brain computer interfaces for enabling locomotion and improving its rehabilitation. The authors consider incorporation of neural technologies in various devices for locomotion, such as wearable lower-limb exoskeletons, orthoses, prostheses, wheelchairs, and assistive-robot devices. The performance of these devices could be improved by BCIs that recognize user intent and provide a communication channel. The EEG communication signals employed by these BCIs are sensorimotor rhythms, event-related potentials and visual evoked potentials. The authors develop a framework for such neural technologies and suggest using this framework for the development of the next generation of intent-based multifunctional controllers for individuals suffering from neuromotor disorders, trauma to nervous system, or limb amputation.

In an opinion article, Bissolotti et al. discuss the limiting factors and evaluate some ethical questions regarding the domestic use of robotic exoskeletons for gait assistance in people affected by spinal cord injury (SCI). While the literature supports the effectiveness of different types of exoskeletons used in a clinical setting to treat such conditions as spasticity, balance impairment and abnormal blood pressure, multiple issues arise when such gait training systems are adapted for the home environments. These issues include high cost of the device and related inequality in the access to health care services, level of competence reached after training to use the device, risk of cardiovascular and metabolic diseases, high risk of osteoporosis of the paretic limbs, skin lesions, deep venous thrombosis and pelvic floor impairment. The article discusses the countermeasures to counteract the negative effects that could increase therapeutic efficacy and decrease inequality in the access to health care services and devices.

## Animal Studies

Several articles report testing neural technologies in animal models of locomotion. In a rabbit amputation model, Millevolte et al. tested and demonstrated the potential for the housing and engagement of a sieve electrode within the medullary canal of long bones as part of an osseointegrated neural prosthesis. Mottaghi et al. investigated cortico-basal ganglia beta oscillations in a rat model of unilateral Parkinson's Disease (PD) and analyzed the effects of deep brain stimulation. They concluded that this model accurately represents many of the motor and electrophysiological symptoms of PD, which makes it a useful tool for pre-clinical testing of new treatments. Park et al. developed a prototype of a powered transtibial prosthesis for the use in a feline model of prosthetic gait. In this prosthesis, the linear actuator operates the prosthetic ankle joint with the control signals that emulate myoelectric activity of muscles; there is a close match between the locomotor patterns produced by the prosthesis and by cats during level walking.

## Balance Control

Three articles address the issue of improving balance control. Buettner et al. describe a virtual balancing apparatus that can modify gravity (e.g., for patients who are unable to balance their full body weight), damping, inertia. The apparatus can be also used to examine the effects of support surface perturbations. It may be well-suited to simulate conditions which could otherwise only be realized in space experiments and to examine the potential benefit for patients of virtual balance training. Sozzi et al. report the effects and the time-course of stabilization produced by a haptic cue provided by a walking cane. They argue that this type of haptic information has many of the features of the direct fingertip contact. They also suggest that the processing time of haptic input from a walking aid should be taken into consideration when designing prostheses for locomotion. Furthermore, Sozzi et al. assess balance improvement in subjects with low vision by adding the haptic input from a cane or fingertip. The authors discuss the ways visual and haptic information could be integrated in in the devices for balance training.

## Prostheses for Locomotion

Three articles analyze available evidence for using robotic devices for the benefit of patients with gait impairments. Malcolm et al. use a biomimetic approach to design prostheses with bi-articular actuation components. In their study, the effect of a bi-articular and spring configuration were tested that mimicked the m. gastrocnemius action; this design was compared to the mono-articular configuration and configuration without a spring. The authors discuss the specific effects of different exoskeleton configurations on metabolic cost and muscle activation that could be useful for providing customized assistance for specific gait impairments. Jayaraman et al. describe the impact of using powered knee-ankle prostheses in two transfemoral amputees. The participants gained the ability to walk with gait kinematics similar to normal gait patterns observed in a healthy limb. These results indicate that powered prosthetic components have a considerable potential to provide safe and efficient gait for individuals with above-the-knee amputation. Finally, van Dijsseldonk et al. report a framework for measuring the effects of training with the Rewalk exoskeleton on the ability to perform basic and advanced skills in people with complete spinal cord injury.

## Myoelectric Control

Myoelectric control of neuroprostheses for walking is a prominent theme in this Research Topic. This is a biologically inspired approach, where electromyography (EMG) feedback from lower-limb muscles assists walking in an exoskeleton or contributes to hybrid rehabilitation systems that combine functional electrical stimulation (FES) and a robotic exoskeleton.

Grazi et al. report a novel assistive control strategy for a robotic hip exoskeleton that relies on the use of the gastrocnemius medialis EMG signal instead of a hip flexor muscle, to control the hip flexion torque and improve the reliability of the control system. Since the presence of moderate to severe spasticity can significantly impair gait kinematics and prevent such individuals from walking in an exoskeleton, Ekelem and Goldfarb applied peroneal stimulation (timed with the exoskeleton swing phase) to acutely suppress extensor spasticity in SCI subjects through the recruitment of the flexion withdrawal reflex. While the common peroneal stimulation had only acute effects (it did not have a significant effect on modified Ashworth scores), it suppressed extensor tone, significantly aided flexion in the hip and ankle joints and enabled improved exoskeletal walking for persons with severe spasticity.

Zhang et al. propose a novel cooperative control strategy, which could realize arbitrary distribution of torque generated by functional electrical stimulation and exoskeleton. Two muscle groups (quadriceps and hamstrings) were stimulated to generate active torque for knee joint in synchrony with torque compensation from the exoskeleton. Experimental evaluation of the hybrid FES-exoskeleton system was conducted in five healthy subjects and four paraplegic patients. The system enabled cooperative control of torque distribution, trajectory tracking, and phase synchronization. Furthermore, Alibeji et al. describe a hybrid neuroprosthesis for walking that combines FES with a powered lower limb exoskeleton. This system has an electrical actuator at the hip and knee joints. The control framework mimics muscle synergy, where FES of the hamstrings and quadriceps muscle group of each leg restores walking in patients with paraplegia. The system was tested in an able-bodied subject and a patient with an incomplete SCI. Another article on myoelectric control (Karunakaran et al.) reports the orthotic and therapeutic effects due to continuous use of a foot drop stimulator (using FES to stimulate the peroneal nerve) in children with foot drop and hemiplegia secondary to brain injury. The authors report that this treatment improves spatial gait asymmetry and increases dorsiflexion and toe displacement during swing. These are a potentially long-lasting effects.

Several articles evaluate the efficiency of the Hybrid Assistive Limb (HAL) exoskeleton, a unique device that performs real-time collection of bioelectric signals from the patient to support and enhance voluntary gait. HAL exoskeletons are equipped with surface EMG electrodes that percutaneously detect minimal bioelectric signals generated by the patient's muscles (hip and knee flexors and extensors). Additionally, floor reaction force signals could be detected caused by patient's intended weight shifts. Sczesny-Kaiser et al. assess the effects and safety of body-weight supported treadmill training with the use of the HAL exoskeleton in patients with limb-girdle muscular dystrophy. All patients involved in this study benefitted from the training. Sczesny-Kaiser et al. report a crossover clinical trial comparing conventional physiotherapy and HAL supported treadmill therapy in chronic stroke patients with hemiparesis. The authors demonstrate the efficiency of individualized therapy based on this approach. Puentes et al. report the effects of HAL therapy in myelopathy (caused by ossification of the posterior longitudinal ligament) patients by performing the principal component analysis of the kinematic data. In this study, HAL therapy improved walking and gait coordination in patients by reshaping their gait pattern. Furthermore, Puentes et al. report an improvement in the intersegmental coordination (assessed by planar covariation of elevation angles) for the paretic and non-paretic side after early HAL intervention in hemiparetic stroke patients. Tan et al. show that muscle synergy analysis can be used as a tool to quantify the change and improvements in neuromuscular coordination of lateral symmetry during gait training in stroke patients with the help of the HAL exoskeleton. Finally, Shimizu et al. describe a case of voluntary ambulation triggered by upper limb activity using the HAL exoskeleton in patients with complete paraplegia after spinal cord injury. The HAL electrodes for the hip and knee flexion-extension were placed on the anterior and posterior sides of the upper limbs contralaterally corresponding to each of the lower limbs. The results suggest that an upper-limb-triggered HAL ambulation is a feasible option for rehabilitation of patients with complete quadri/paraplegia caused by chronic SCI.

## Electroencephalography (EEG)-Based Systems for Controlling Lower Limb Prostheses

A group of articles report and analyze the usage of EEG signals for the control of prostheses for locomotion. Murphy et al. assess the benefits of using an EEG-based BCI for lower-limb prostheses. The feasibility of such systems is supported by the implementation of a reliable knee-locking switch based on a EEG rhythm-feedback training for swing phase during walking and for sitting down in a transfemoral amputee. Ortiz et al. describe a new tool based on the Stockwell transform for the analysis of the EEG signals during gait cycle. Extraction of instantaneous EEG characteristics with this method was successfully tested in healthy individuals and patients with lower limb disabilities. Mejia Tobar et al. examine the classification of ankle flexion and extension movements from cortical current sources estimated by a hierarchical variational Bayesian method they use EEG and fMRI recordings. The presented method is also applicable to real-time BCIs and has the potential to identify neural patterns to control exoskeletons, prostheses and functional electrical stimulators. Wei et al. investigate the feasibility of gait phase recognition based on EEG combined with EMG using the corticomuscular interaction analysis and the time–frequency cross mutual information method. They report good recognition for three different walking speeds and discuss a theoretical basis for gait recognition based on EEG and EMG recordings during patient rehabilitation with lower limb exoskeletons.

## Combined Effects of Spinal Cord Neuromodulation and Exoskeleton Walk Training in Paralyzed Individuals

Finally, a promising approach is based on neuromodulation of the spinal circuitry during walking in the exoskeleton. This method is particularly beneficial for gait rehabilitation after SCI. This approach makes use of the activation of central pattern generation circuits that largely depend on the presence of a sustained excitatory drive (as it can be elicited by non-invasive transcutaneous electrical spinal cord stimulation either without or with specific pharmacological neuromodulation) combined with the over-ground step training in an exoskeleton. In particular, Gad et al. in a case study report that spinal cord stimulation combined with pharmacological treatment enhanced the level of effort and improved the coordination patterns of the lower limb muscles, resulting in a continuous stepping motion in the exoskeleton along with the improvements in autonomic functions including cardiovascular and thermoregulation. In a large cohort of SCI patients, Shapkova et al. show that percutaneous electrical stimulation of the lumbar enlargement and exoskeleton-induced walking work together well to assist walking in SCI patients. Particularly, anti-spastic stimulation at high frequency enabled individuals with severe spasticity to be able to use the exoskeleton for walking. Overall, this study showed that the 2-week intensive synergistic effect of exoskeleton-assisted walking and the simultaneous spinal cord stimulation improve gait and neurological signs in chronic SCI, including complete paralysis.

Taken together, this Research Topic demonstrates the growth of interest to using neural prostheses for the restoration of locomotion in patients with neurological disorders that impair gait.

## Author Contributions

All authors listed have made a substantial, direct and intellectual contribution to the work, and approved it for publication.

## Funding

This work was supported by the Russian Science foundation Grant 21-75-30024, USA National Science Foundation (NSF) Grant # 1912557, and the Italian Ministry of Health (Ricerca Corrente, IRCCS Fondazione Santa Lucia).

## Conflict of Interest

The authors declare that the research was conducted in the absence of any commercial or financial relationships that could be construed as a potential conflict of interest.

## Publisher's Note

All claims expressed in this article are solely those of the authors and do not necessarily represent those of their affiliated organizations, or those of the publisher, the editors and the reviewers. Any product that may be evaluated in this article, or claim that may be made by its manufacturer, is not guaranteed or endorsed by the publisher.

